# *Propionibacterium acnes*: An Underestimated Pathogen in Implant-Associated Infections

**DOI:** 10.1155/2013/804391

**Published:** 2013-11-06

**Authors:** María Eugenia Portillo, Stéphane Corvec, Olivier Borens, Andrej Trampuz

**Affiliations:** ^1^Microbiology Laboratory, Laboratori de Referencia de Catalunya, Barcelona, Spain; ^2^Service de Bactériologie-Hygiène, CHU de Nantes, Institut de Biologie, Nantes Cedex, France; ^3^Université de Nantes, EA3826, Thérapeutiques Cliniques et Expérimentales des Infections, 1 rue G. Veil, 44000 Nantes, France; ^4^Orthopedic Septic Surgical Unit, Department of Surgery and Anesthesiology, Lausanne University Hospital, Lausanne, Switzerland; ^5^Center for Musculoskeletal Surgery, Charité-University Medicine Berlin, Free University and Humboldt University, Charitéplatz 1, 10117 Berlin, Germany

## Abstract

The role of *Propionibacterium acnes* in acne and in a wide range of inflammatory diseases is well established. However, *P. acnes* is also responsible for infections involving implants. Prolonged aerobic and anaerobic agar cultures for 14 days and broth cultures increase the detection rate. In this paper, we review the pathogenic role of P. acnes in implant-associated infections such as prosthetic joints, cardiac devices, breast implants, intraocular lenses, neurosurgical devices, and spine implants. The management of severe infections caused by *P. acnes* involves a combination of antimicrobial and surgical treatment (often removal of the device). Intravenous penicillin G and ceftriaxone are the first choice for serious infections, with vancomycin and daptomycin as alternatives, and amoxicillin, rifampicin, clindamycin, tetracycline, and levofloxacin for oral treatment. Sonication of explanted prosthetic material improves the diagnosis of implant-associated infections. Molecular methods may further increase the sensitivity of *P. acnes* detection. Coating of implants with antimicrobial substances could avoid or limit colonization of the surface and thereby reduce the risk of biofilm formation during severe infections. Our understanding of the role of *P. acnes* in human diseases will likely continue to increase as new associations and pathogenic mechanisms are discovered.

## 1. Introduction

*Propionibacterium acnes* is part of the normal human microbiota [1, 2]. This bacterium is usually responsible for late chronic infections but, exceptionally, could produce acute infections, mainly related to any device. The *P. acnes* genome encodes diverse virulent factors which confer a pathogenic potential to this bacterium [3].

**Figure 1:**
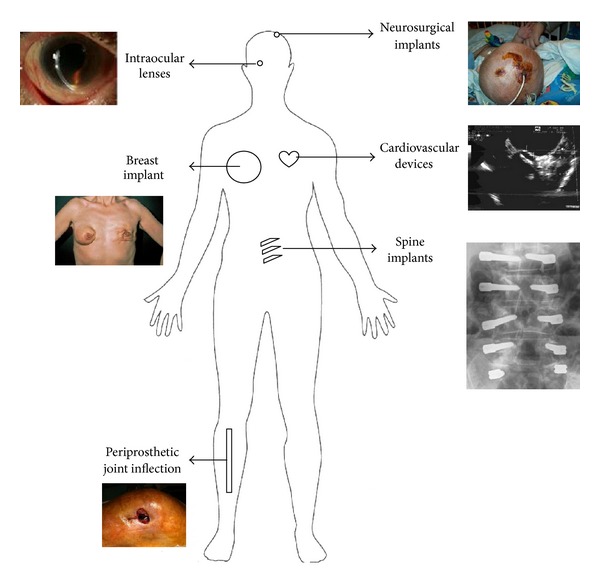
Diversity of implant-associated infections caused by *P. acnes*.

The role of *P. acnes *in the pathogenesis of acne is known for decades. Numerous reports reveal that *P. acnes* has been also associated with chronic prostatitis leading to prostate cancer [4], chronic recurrent multifocal osteomyelitis (CRMO) and synovitis-pustulosis-hyperostosis and osteitis (SAPHO) syndrome [5], sarcoidosis [6, 7], and sciatica [8]. More recently, this microorganism has been recognized as the cause of various types of implant-associated infections, including breast implants [9, 10], neurosurgical shunts [11], cardiovascular devices [12], ocular implants [13], internal fracture fixation devices, spinal hardware [14], and prosthetic joints [15] (Figure 1).

## 2. Microbiology

**Figure 2:**
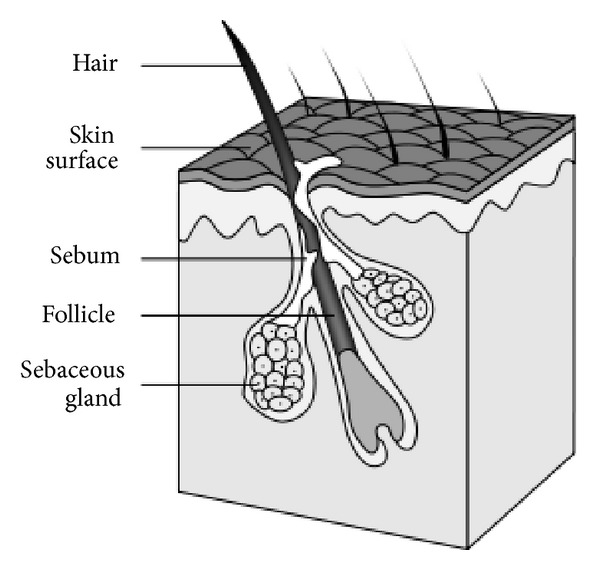
Scheme of a normal pilosebaceus unit of human skin. The hair, sebum, and keratinocytes that fill the narrow follicle may produce a plug. The mixture of oil and cells desquamate allows *P. acnes* to grow in the plugged follicles, producing chemicals and enzymes that attract host immune cells causing inflammation. Source: National Institutes of Health (NIH), Department of Health and Human Services.

*P. acnes* is an anaerobic-aerotolerant diphtheroid-like Gram-positive bacillus that resides in pilosebaceous follicles of the skin (Figure 2) [1], and is also found in the conjunctiva [2], oral cavity [2], intestinal tract [16], and external ear canal [1]. The *P. acnes* genome encodes all key components of oxidative phosphorylation (NADH dehydrogenase/complex I, cytochrome c reductase, cytochrome c oxidase, and FOF1-type ATP synthase). In addition, it also possesses the genes for the cytochrome d oxidase, which ensures growth in different conditions [3, 17, 18]. Therefore, *P. acnes* can tolerate exposure to oxygen for several hours and is capable *in vitro* to survive under anaerobic conditions for up to 8 months [19]. The latter observation suggests that *P. acnes* can also survive for a prolonged period in human tissues with low oxidation potential [18, 19]. 

Despite its oxygen-tolerant characteristics, *P. acnes* is not reliably detected by aerobic culture due its slow growth [20]. The optimal temperature for growth is 37°C. To increase the detection, prolonged aerobic and anaerobic agar cultures to 14 days and inoculation into thioglycollate broth should be routinely performed [20–22]. In particular, the low redox potential of enriched thioglycollate broth supports growth of *P. acnes*. Importantly, thioglycollate broth should be routinely subcultured on agar plates despite the absence of visible turbidity of the broth medium [23]. However, a positive culture with *P. acnes* should be interpreted with caution. For example, in case of recovery in broth cultures only or from only one of several tissue samples, additional criteria of infection (such as clinical signs, positive histopathology, or molecular tests) are required [23, 24]. In addition, *P. acnes* should be considered as pathogen in chronic or persistent low-grade implant-associated infections without positive cultures, in which this pathogen is probably underrecognized and underestimated [25, 26].

According to serological agglutination tests and cell wall carbohydrate analysis, *P. acnes* can be classified into two different phylotypes (type I and II) [27, 28]. By sequence analysis of two genes, a nonribosomal housekeeping gene (*recA*) and a gene encoding a putative hemolysin/cytotoxin (*tly* gene), further discrimination into phylogenetically distinct clusters (type IA, IB, IC, II, and III) is possible [29]. Recently, MALDI-TOF Mass Spectrometry seems to be a useful tool for rapid identification and typing of *P. acnes* [30]. Several genes in the *P. acnes* genome encode different virulent factors such as hemolysins, CAMP factors, lipases, esterases, surface-associated proteins, or cellular factor with antigenic potential [3, 31]. *P. acnes* also encodes genes with poly (C)/(T) stretches. Such variations seem to be involved in phase variation, an adaptation strategy to evade immune responses and degradation [3]. However, from infected prosthesis type IB strains were more frequently isolated than type IA.

Several researchers investigated the association between the pathogenicity of different *P. acnes* phylotypes and their clinical significance [3, 32, 33]. No clear association between phylotypes and infection/colonization has been found. Nevertheless, some phylotypes have been described more frequently in specific infections than others. For instance, in a recent study on shoulder implant infections, *P. acnes* was isolated only in male patients, suggesting that host factors could predispose for infection with this microorganism [34]. Moreover, *P. acnes* type I was predominant in all types of orthopedic implants, except in prosthetic hip joints, in which type I and type II showed equal frequency. 

Genomic variation among individual types seems to be low, but there are key differences in genomic island-like regions encoding a variety of virulence-associated traits [32]. Biofilm formation may be one virulence determinant facilitating implant-associated infections [3]. The genome sequence has revealed three clusters of genes that encode enzymes involved in extracellular polysaccharide biosynthesis and adhesion proteins required for biofilm production [3]. 

## 3. Implant-Associated Infections

### 3.1. Periprosthetic Joint Infection

The pathogenesis of periprosthetic joint infection (PJI) is related to microorganisms growing in biofilms, rendering these infections difficult to diagnose and to eradicate [35–37]. PJI can be caused by direct contamination of the surgical wound and the implant during surgery (i.e., perioperative infection), by spreading from a remote infectious focus (i.e., hematogenous infection) or by extension from a neighbored focus or penetrating injury (i.e., contiguous infection) [37]. Staphylococci are the most commonly isolated organisms (*≈*50%), followed by streptococci (*≈*10%), enterococci (*≈*10%), and Gram-negative bacilli (*≈*10%) [37, 38]. The frequency of *P. acnes* is reported in approximately 10% of PJI, but its frequency is most likely underestimated due to short incubation times in many routine laboratories and its growth being considered as contamination [23]. *P. acnes* is most frequently associated with shoulder PJI and spine implant-associated infections due to a high concentration of sebaceous follicles at these sites [15, 39]. 

Low-grade infections are typically manifested 3 to 24 months after implantation, or occasionally up to 36 months or longer. As *P. acnes* belongs to the normal skin microbiota, the significance of its growth may be difficult to determine [23]. In low-grade infection, the values of systemic inflammatory biomarkers, such as C-reactive protein (CRP), are often normal [40–42]. Similarly, periprosthetic tissue histopathology may show no acute or only chronic inflammation, reflecting the low virulence and low bacterial burden of *P. acnes* [40, 43]. A combination of various preoperative and intraoperative tests is needed for accurate diagnosis of low-grade infection of prosthetic joints [23, 44]. When PJI is diagnosed, a two-stage exchange is usually performed since the prosthesis is typically loosened and retention is not possible anymore. A short interval between prosthesis explantation and reimplantation (i.e., 2-3 weeks) is increasingly used, if *P. acnes* is susceptible to rifampin, the key antibiotic against biofilms [45]. 

Current diagnostic methods for PJI such as periprosthetic tissue cultures have limited sensitivity, with 10–20% false-negative cultures [46]. Recently, molecular techniques have been developed to increase the sensitivity of PJI detection, but *P. acnes* specific primers are not included in such assays [46, 47]. 

Several investigators suggested that some cases of prosthesis failure considered as aseptic may actually be of infectious etiology [48–50]. However, the survival time for joint prosthesis was not shorter when *P. acnes* was cultured in aseptic loosening cases (about 20% of cases), suggesting that this microorganism may have another, yet unknown, role in such cases [51]. 

### 3.2. Cardiac Device Infection

*P. acnes* infective endocarditis remains rare, although its prevalence is probably underestimated due to diagnostic difficulties [25]. Infection mostly involves prosthetic heart valves [25], annuloplasty rings [52], and pacemaker/ICD leads [53]. Bacteremia or skin wounds are the most frequent port of entry of microorganisms [12]. *P. acnes* infective endocarditis often develops on valve prostheses and embolisms are common. To the best of our knowledge, less than 50 cases of infective endocarditis have been described on prosthetic heart valves, usually the aortic valve prosthesis [25]. Due to subtle symptoms and slow growth of the microorganism, the diagnosis is often late, when valvular and peri-valvular destruction is significant [54]. Antibiotic therapy and surgical intervention with change of the valve are typically needed, and the mortality is high (15–27%) [12, 55]. The diagnosis of *P. acnes* infective endocarditis using Duke criteria is challenging [56], since echocardiography can be normal and the dysfunction progresses slowly over weeks and months leading to cardiac insufficiency [25, 57, 58]. Fever appears only in approximately 25% of these patients, and the incidence of neurologic symptoms is higher than that in general complication of infective endocarditis [25, 55]. 

### 3.3. Breast Implant Infection

Breast implants are increasingly used for aesthetic reasons or in patients after mastectomy [59, 60]. Infection occurs in 1.1% to 2.5% after aesthetic breast augmentation and up to 35% after breast implant reconstruction following mastectomy [61]. These infections are typically caused by bacterial skin flora, such as *Staphylococcus aureus* and coagulase-negative staphylococci [62]. Acute infections associated with breast implants usually occur during the first month after implantation and are frequently associated with fever, acute pain, and marked breast erythema. In some cases, *P. acnes* could be recovered alone or in combination with staphylococci. Late infections are rare and often associated with bacteremia or an invasive procedure at a location other than the breasts. Risk factors for breast implant infection are breast reconstruction after mastectomy and radiotherapy [62]. Surgical removal of the implant is mandatory in most cases by two-step procedure [63].

**Figure 3:**
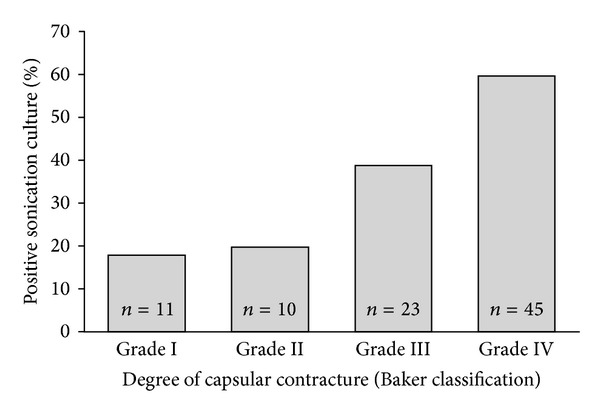
High correlation between the degree of capsular contracture and sonication culture of 89 removed breast implants without clinical signs of infection (*P* for trend <0.001). Reproduced with permission from Rieger et al. [71].

Developments in chemistry have improved the material characteristics of breast implants and reduced capsular contracture [64]. However, the incidence of capsular contracture after breast implant surgery is up to 30% and its etiology remains unclear [65, 66]. The modified Baker classification of capsular contracture includes degree I to IV [67]. Possible explanations for this important complication include chemical interference of the implant in the surrounding tissues, mechanical impact by the anatomical position of the implant, and the effect of bacteria growing in biofilms on the implant surface [68]. Another possible cause of capsular contracture is the presence of bacteria growing as biofilm on the surface, which may cause persistent low-grade inflammation of the surrounding tissue, leading to formation of capsular fibrosis and subsequent contracture [69, 70]. A significant correlation between the degree of capsular contracture and the presence of biofilm on breast implants was demonstrated by several authors, especially when using a sonication technique [9, 10, 71]. In a recent study, 112 breast implants were sonicated. Fifty two of them had a positive sonication fluid culture (46%). Among positive sonication fluid cultures, *P. acnes* was isolated in most cases (54%). *P. acnes* seems to have a role especially in Baker grade IV (Figure 3) [72]. Its origin is most likely the patient skin or colonized mammary ducts at the incision site [62].

### 3.4. Infections of Intraocular Lenses

Infectious endophthalmitis is the most devastating complication of intraocular surgery. The incidence of infection after cataract surgery and posterior chamber lens implantation is low, reported from 0.07 to 0.33% [73]. Bacterial biofilm is produced in the intraocular lenses by microorganisms which adhere to the lenses [74]. Postoperative endophthalmitis can be classified into acute and delayed infection. While acute endophthalmitis usually occurs soon after surgery and is caused by *S. aureus*, *Streptococcus* spp., or coagulase-negative staphylococci, delayed infections appear from months to years after surgery and are predominantly caused by low-virulent microorganism such as *P. acnes*, *Actinomyces* spp. or *Corynebacterium* spp. [75]. Endophthalmitis diagnosis is based on the appearance of ocular signs and symptoms combined with microscopic and microbiologic examination of the intraocular samples [76]. The most characteristic manifestation of delayed postoperative endophthalmitis is the appearance of white plaques on the lens capsule or the intraocular lens associated with chronic, recurring intraocular infection [75]. Cultures of vitreous biopsy sample often fail to detect the causative microorganisms due to their low number and low virulence in delayed postoperative endophthalmitis [76]. Molecular diagnostic techniques by PCR have several advantages, although they are not always routinely implemented [75]. The surgical approach, especially in *P. acnes* cases, involves a pars planavitrectomy and may include a selective posterior capsulectomy with intraocular injection of antibiotics or a total capsulectomy with intravitreous antibiotics as well as extraction of the intraocular lens [77]. 

### 3.5. Neurosurgical Shunt Infection

*P. acnes* is increasingly documented in neurosurgical infection involving internal cerebrospinal fluid (CSF) shunts and external ventricular drains (EVD). Internal shunts generally transfer CSF into the peritoneal cavity (ventriculo-peritoneal shunts) but can be placed also into the right atrium (ventriculo-atrial shunts) or rarely in the pleural space, ureter, gall bladder, or fallopian tube [78]. Internal CSF shunt infection constitutes a serious complication with considerable mortality and morbidity, especially in pediatric patients [79]. The infection rate ranges from 1.5 to 38% [80]. 

Shunt infections can be classified as early or late depending on whether these infections occur before or after the first year of surgery [79]. Early shunt infections are mainly caused by skin microorganisms introduced during surgery [81]. Late shunt infections are less frequent, <1% annually [82], and usually related to peritonitis (generally due to appendicitis) or to hematogenous sources from secondary infections [83]. Clinical manifestations include fever, shunt malfunction, malaise, poor feeding, peritoneal signs, localized abdominal abscess/CSF collection, and wound breakdown [83, 84]. In the absence of fever, differentiation between shunt dysfunction and shunt infection is difficult [20, 85]. Moreover, allergic reactions to shunt material such as silicone or ethylene oxide may also mimic shunt infections [86, 87]. A young age at the time of initial shunt placement and a short time interval from previous surgical revision are risk factors associated with shunt infection [88]. In general, coagulase-negative staphylococci, *S. aureus*, and *P. acnes* are the most common infecting microorganisms implicated in shunt infections [89]. Although *P. acnes* shunt infections are mainly triggered by bacterial contamination from the skin during surgery, symptoms may occur weeks to years after shunt placement or manipulation [20]. 

Shunt infection is diagnosed based on the combination of clinical signs, CSF cell count and CSF culture sampled through a reservoir tap or lumbar puncture [89]. Differentiation between true infections and contaminations remains difficult. Visualization of microorganisms after Gram straining of CSF is often not possible, and cellular and chemical fluid changes may be subtle [20]. *P. acnes* shunt infections typically are indolent and present with normal serum CRP levels [90]. Patients present with low initial leukocyte count, percentage of neutrophils, high peak of eosinophil percentage, and minor changes in CSF including glucose or protein levels [20, 91]. However, CSF eosinophilia could be associated with reactions to foreign substances, particles or blood, and obstruction of tubing but also by infection caused by coagulase-negative staphylococci [92]. Therefore, culture of CSF remains the most valuable tool for the diagnosis of shunt-associated infection [20]. 

The most effective treatment for *P. acnes* shunt infection is shunt removal, temporary placement of an external ventricular drainage or ventricular taps (if needed), and treatment with high-dose intravenous penicillin G [20, 93]. Retention of the distal shunt part often leads to relapse of infection and shunt failure [94]. A new shunt should be placed when CSF becomes sterile [94]. Antibiotic-impregnated shunt systems were introduced to prevent shunt infection [80]. Shunt catheters can be impregnated with antibiotics (often rifampicin or clindamycin). By using these catheters with antibiotics, there is a risk of an increase in the rate of antibiotic resistance rate due to selective pressure. In a recent study, the incidence of CSF shunt infection was lower in patients with antibiotic-impregnated shunt systems compared with those without [95]. 

External ventricular drains (EVDs) are used in acute hydrocephalus to prevent further brain damage due to high CSF pressure. The rate of external ventricular drains-associated infection ranges from 5 to 22% in high-risk patients [96–98]. Factors associated with increased risk of infection are intraventricular or subarachnoid hemorrhage, cranial fracture with CSF leakage, and catheter irrigation [96, 99–102]. Although several studies demonstrated that prolonged external ventricular drains-indwelling time (>3 to 5 days) constitute a risk factor for external ventricular drains-associated infection [98, 103–106], it remains unclear whether a regular external ventricular drains exchange can reduce the infection risk [99, 107–110]. EVD-associated infection may occur up to 10 days after removal of EVD. Clinical signs and symptoms of external ventricular drains-associated infections are nonspecific, such as fever and headache, and often overlap with signs and symptoms of the underlying neurosurgical condition. In addition, CSF parameters may vary widely and none has been shown to be predictive for infection, nor cutoff values have been established [96, 99, 111, 112]. A positive Gram stain or CSF culture are highly specific for external ventricular drains-associated infection but not very sensitive [113, 114]. Cultures of the external ventricular drains tips may increase the sensitivity, especially when the removed catheter is sonicated. Most authors suggest performing a complete CSF diagnostic workup if EVD-associated infection is suspected, including CSF leukocyte count and differential, as well as CSF Gram stain and culture [104, 115, 116]. In a recent study, although most commonly isolated organisms causing EVD-associated infection were coagulase-negative staphylococci (63%), *P. acnes* represented 15% of the cases [117].

### 3.6. Spine Implant Infection

The rate of infection after spinal surgery is low, about 0.2%; however, this rate increases up to 12% when instrumentation is used [118–120]. Nevertheless, the microbiologic diagnosis of spinal implant infection can be challenging. Spinal implant infections can be classified as early or late depending on whether the infection occurs before or after the first month of surgery [121]. Although *P. acnes* and *S. epidermidis* are the most common bacterial causes of late postoperative infection [39], *P. acnes* is also related to 3–50% of early postoperative infections [121, 122]. Clinical manifestations of spine implant infection are usually nonspecific like back pain and paravertebral spasms [123]. Other common symptoms include drainage and localized swelling or fullness along the length of the incision [119, 120]. Fever is infrequently reported [123]. Inflammatory biomarkers such as CRP, ESR, and WBC values are unreliable as diagnostic markers of low grade spinal implant infections because they may be within the normal range [121]. Presence of infection is usually confirmed by radiographic and microbiological findings. Magnetic resonance imaging (MRI) is considered as the best diagnostic imaging approach (when no device is present) to detect spinal infections [123]. The causative pathogen can be isolated by culturing samples taken by biopsy or peri-implant tissues [124]. Implant sonication is more sensitive than peri-implant tissue culture [39]. The management of spinal implant infection is controversial. While some authors recommend serial debridement surgeries with implant retention [125, 126], others advocate implant removal [127]. Contraindications to hardware removal are stated in cases where fusion has not yet taken place [128]. Improved infection-free interval has been reported using long-term oral suppressive antibiotics [125]. 

## 4. Treatment

The management of severe infections caused by *P. acnes* involves a combination of intravenous antimicrobial agents and surgical procedures (e.g., removal of the device and/or debridement of the surgical site). For serious infections, penicillin G and ceftriaxone are considered antibiotics of first choice [129], with vancomycin and daptomycin as alternatives in case of *β*-lactam allergy or antimicrobial resistance. Clindamycin, tetracycline, and levofloxacin are oral alternatives for nonserious infections, mostly skin diseases [130]. Rifampin is considered active against *P. acnes* biofilm [131]. Importantly, *P. acnes* is intrinsically resistant to metronidazole and fosfomycin. Aminoglycosides have generally weak activity and should not be used in the treatment of *P. acnes* infections. 

## 5. Outlook

Sonication of explanted prosthetic material has shown to be more sensitive than conventional microbiological culture in the diagnosis of foreign body infections, especially in orthopedic prosthesis, breast implant, and cardiac devices [10, 132, 133]. Formation of *P. acnes* biofilms on implants highlights the importance of vortexing/sonication to detach the microorganism prior to culture [134, 135]. Therefore, sonication procedure should be applied routinely to all types of implants in order to improve the diagnosis of implant-associated infections caused by *P. acnes*. Also, disruption of the periprosthetic tissue samples improves the diagnosis as adherent cells are detached and removed [136]. Otherwise, new molecular methods for implant-associated infections may be developed in the future to provide rapid and sensitive detection of *P. acnes*. Moreover, these techniques are not affected by previously administered antibiotics. These new techniques would also be able to detect virulence or antibiotic resistance genes and specific mRNA to differentiate active from latent or previous infection. Coating of implants with antimicrobial substances may avoid colonization of the surface by microorganisms and reduce the risk of biofilm formation and clinical infections. Our understanding of the role of *P. acnes* in human diseases will likely continue to increase as new associations and pathogenic mechanisms will be discovered.
